# Pleural effusion as the initial extramedullary manifestation of Acute Myeloid Leukemia

**DOI:** 10.12688/f1000research.1-39.v1

**Published:** 2012-10-30

**Authors:** José Nieves-Nieves, Luis Hernandez-Vazquez, Dev Boodoosingh, Ricardo Fernández-Gonzalez, Rosángela Fernández-Medero, José Adorno-Fontánez, Edgardo Adorno-Fontánez, José Lozada-Costas

**Affiliations:** 1 Pulmonary Medicine, San Juan City Hospital, San Juan, Puerto Rico; 2 Internal Medicine, San Juan City Hospital, San Juan, Puerto Rico; 3 Hematology-Oncology Medicine, San Juan City Hospital, San Juan, Puerto Rico

## Abstract

Leukemias rarely debut by pleural involvement as the first manifestation of the hematologic malignancy. This complication is most commonly seen in solid tumors such as carcinomas of the breast, lung, gastrointestinal tract and lymphomas. We present a case of a 66 year old male who presented with a pleural leukemic infiltration of his undiagnosed Acute Myeloid Leukemia that was not a complication of the disease extension, but the acute presentation of the illness. Progressive shortness of breath for two weeks, cough, clear sputum and weight loss were the initial complaints. Serum dyscrasia suggested a hematologic abnormality. A chest x-ray performed demonstrated a buildup of fluid with layering in the left pleural cavity. Diagnostic thoracentesis suggested an exudative etiology with cytology remarkable for 62% leukemic myeloblast. The diagnosis was confirmed by bone marrow biopsy with expression of the antigens CD 34+ and CD13+, with unfavorable cytogenetic prognosis and a trisomy 21 chromosomal defect. Chemotherapy was initiated, though no remission achieved with induction chemotherapy. Complications and disease progression precludes in the patient’s death. Although rare, due to the unusual presentation of the disease, this case clearly demonstrates the importance of biochemical analysis and cytopathology specimens obtained in pleural fluid.

## Introduction

Acute Myelogenous Leukemia (AML) is a group of hematogenous neoplasms characterized by clonal proliferation of myeloid precursors with a reduced capacity to differentiate into more mature cellular elements ^1^. As a result, there is an accumulation of leukemic blasts or immature forms in the bone marrow, peripheral blood, and occasionally in other tissues, with a variable reduction in the production of normal red blood cells, platelets, and mature granulocytes. The increased production of malignant cells, along with a reduction in these mature elements, result in a variety of systemic consequences including anemia, bleeding, and an increased risk of infection ^1^. Less than 1 percent of patients present with prominent extramedullary disease ^2^. These extramedullary manifestations can manifest simultaneously with, or precede, bone marrow involvement. Sites of isolated expression include bone, periosteum, soft tissues, and lymph nodes, and less commonly the orbit, intestine, mediastinum, epidural region, uterus, and ovary ^2^. To our knowledge this is one of the few reported cases of pleural effusion as the initial manifestation of AML.

## Case report

A 66 year old man with a long-standing history of mild to moderate asthma and arterial hypertension was evaluated for a worsening productive cough of clear sputum, dyspnea, wheezing, and unintentional weight loss of approximately thirty pounds. The patient denied fever, chills, hemoptysis, night sweats, chest pain, or exposure to sick contacts. His medications were frequent use of short acting β-agonist with minimal resolution of symptoms.

On physical examination, the patient was alert but in mild respiratory distress, afebrile without hemodynamic compromise. The cardiac examination was normal; pulmonary examination revealed diffusely decreased breathing sounds, inspiratory crackles, and dullness to percussion, decreased fremitus and egophony in up to two thirds of the left lung field. There was no use of accessory muscles and oxygen saturation was 90% with the patient breathing ambient air. Neither lymphadenopathy nor organomegaly was palpated. CBC was abnormal for hemoglobin 8.1 g/dL, platelet 60,000/µL, leukocyte count 87000/µL with 64% blast ( Table 1). Arterial blood gases were pH 7.402, PCO _2_ 38.3 mmHg, and PO _2_ 67 mmHg; oxygen saturation was 89% without supplemental oxygen.

**Table 1.** Complete blood count with differential.

Laboratory studies	Results
Hemoglobin	8.1 g/dL (81 g/L)
Platelets	60,000/µL (60 × 10 ^9^/L)
Leukocyte	87,000/µL (87 × 10 ^9^/L)
Blast	64%
Neutrophils	10%
Lymphocytes	14%

**Figure 1.**
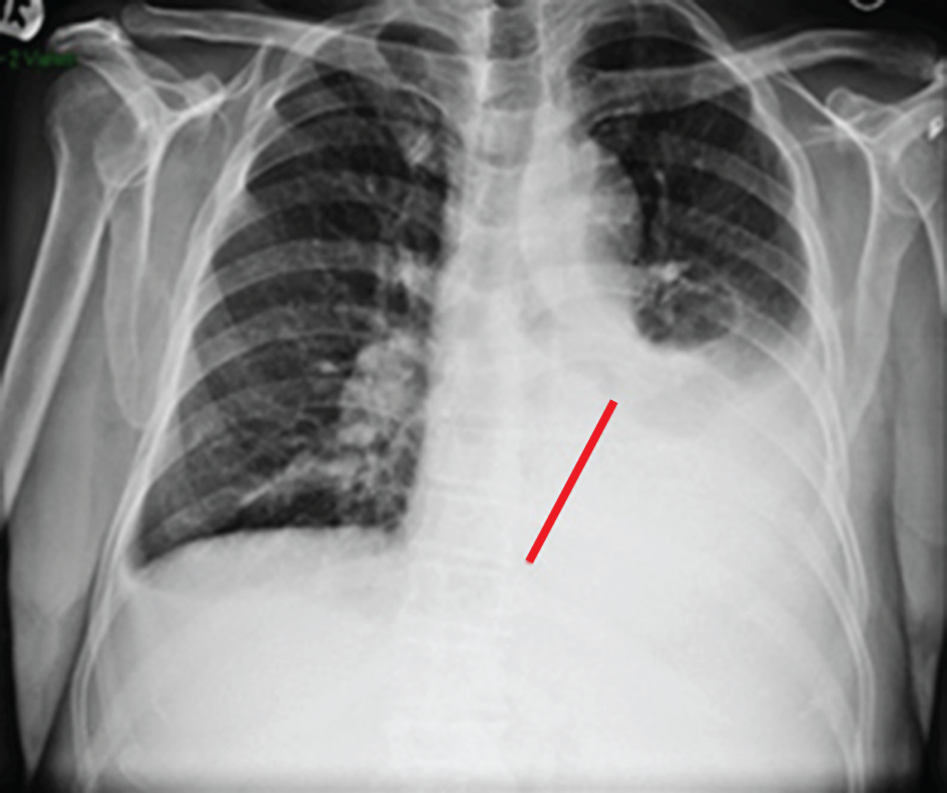
CXR with left pleural effusion (red bar).

**Figure 2.**
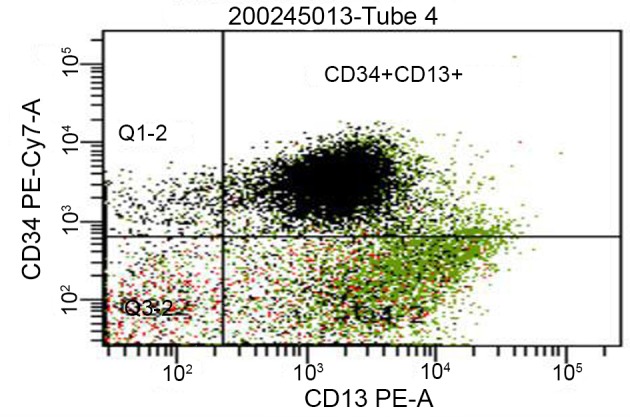
Flow cytometric quantification and immunophenotyping of leukemic stem cells in our patient with acute myeloid leukemia demonstrating expression of CD 34+ and CD13+ antigens on immature cells.

A hematologic malignancy was suggestive due to the serum dyscrasia. Chest radiograph showed a large free flowing left pleural effusion ( Figure 1). A diagnostic and therapeutic thoracentesis was performed with removal of approximately 1 liter of fluid. The symptoms resolved and biochemical analysis established an exudative etiology ( Table 2). The cytopathology specimen obtained from the pleural fluid was positive for blast cells with 62% leukemic myeloblast. AML was confirmed by bone marrow biopsy with expression of the antigens CD 34+ and CD 13+ ( Figure 2) with intermediate to unfavorable cytogenetic prognosis ( Table 3).

**Table 2.** Pleural fluid description. Ratio of the pleural fluid lactate dehydrogenase and protein to serum lactate dehydrogenase and protein (Light’s criteria meeting exudative etiology).

Color	Protein	LDH	Glucose	pH	PF _protein_/ Serum _protein_	PF _LDH_/ Serum _LDH_
Dark yellow	5.4	537	88	7.5	0.74	1.09

**Table 3.** Flow cytometry differential of leukocyte population demonstrating low immunophenotypic values of lymphocytes and granulocytes which demonstrates an unfavorable cytogenetic prognosis.

Flow cytometry differential (% of Total cells)	
Lymphocytes	2
B-cells	<1
Kappa	<1
Lambda	<1
Kappa:Lamda Ratio	1
T-cells	1
CD4	1
CD8	1
CD4:CD8 Ratio	1.6
CD3+CD56+	<1
Natural killer cells	1
Monocytes	7
Granulocytes	20
CD34-Positive blasts	62
Plasma cells	<1
Viability	99

**Figure 3.**
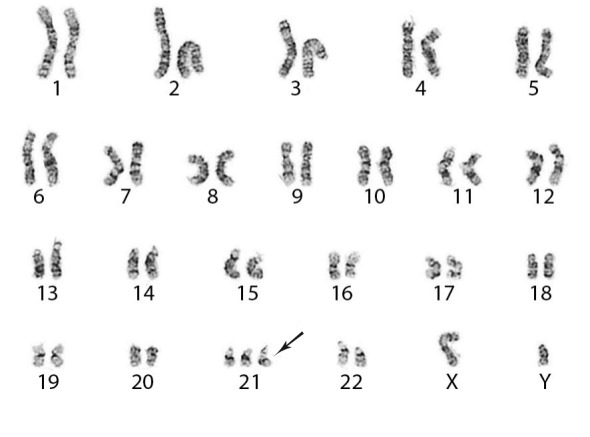
Trisomy 21 as the sole acquired karyotypic abnormality in our patient with acute myeloid leukemia (arrow).

A karyotypic abnormality of Trisomy 21 was revealed through cytogenetic studies ( Figure 3), which is the second most common chromosomal defect in AML. The patient was treated with Idarubicin combined with Cytarabine for the recently discovered AML and there was no re-accumulation of the pleural fluid. However, bone marrow aspiration was repeated to assess response to chemotherapy and he still presented with 62% of blasts cells. A new cycle of chemotherapy was started with Mitoxantrone, Etoposide and Cytarabine but only a partial response was obtained. Despite the therapeutic regimen, due to the severity of the disease and the poor cytogenetic prognosis, the patient’s condition deteriorated. In view of no significant response to therapy and dismal prognosis, supportive measures and palliative care was provided and eventually the patient died due to complications associated to AML.

## Discussion

Physicians dealing with the diagnostic workup of pleural effusions rarely discover an underlying hematologic malignancy ^1^. AML generally presents with symptoms related to complications of pancytopenia. Most patients have more subtle evidence of bone marrow involvement for weeks, or perhaps months, before the diagnosis can be made. Despite the pancytopenia, and/or coagulopathy, it is unusual for leukemias, either acute or chronic, to manifest with malignant pleural effusions as the initial presentation ^3– 5^. Usually this abnormal amount of fluid collection is a complication more commonly seen in solid tumors and lymphomas ^5^.

Our patient presented with AML and pulmonary involvement with signs and symptoms secondary to the pleural effusion itself rather than with classical appearance of the acute myeloid leukemia. An unusual case where neither the complications of the hematologic dyscrasia such as bleeding and recurrent infections, nor the physical findings of a swollen spleen, liver, or lymph nodes were the primary target organs that would lead to a presumptive diagnosis. This demonstrates the importance of the biochemical analysis and the cytopathology specimens obtained in pleural fluid since an early detection of any determined disease could guide effective therapy ^6, 7^. AML in this particular case, and prompt treatment could undoubtedly contribute in avoiding complications associated with the condition; an essential factor for improving quality of life. For this reason chest physicians should be aware of all possible pulmonary manifestations of hematologic malignancies.

## Consent

Written informed consent for publication of clinical details and clinical images was obtained from the relative of the patient.
