# Supplementary material for: Pleural effusion as the initial extramedullary manifestation of Acute Myeloid Leukemia
Source: F1000Res. 2012 Oct 30;1:39. [Version 1] doi: 10.12688/f1000research.1-39.v1 (PMC3814911; doi:10.12688/f1000research.1-39.v1)
Supplement: Peer review history

# Peer review: Referee response for version 1

**Published:** 2012-11-29

**Authors:** Adlette Inati

**Affiliations:** 1 Division of Pediatric Hematology and Oncology, Children's Center for Cancer and Blood Diseases, Rafik Hariri University Hospital, Beirut, Lebanon

The authors report a case of pleural effusion as an initial presentation of Acute Myeloid Leukemia (AML) in an adult patient with no physical exam findings suggestive of leukemia and or malignancy.

The authors corroborated the exudative nature of this symptomatic s pleural effusion by flow cytometric analysis for CD34+ and CD13+ antigens and supported their diagnosis by bone marrow studies and cytogenetic analysis which showed a trisomy 21 pattern. While a chemotherapeutic regimen of Cytarabine and Idarubicine inhibited further progression and re accumulation of the pleural effusion, the patient’s condition deteriorated leading to his death. Given the dismal nature of the patient’s AML prognosis, hematologists need to be aware of this atypical and rare AML presentation. More importantly, pulmonary physicians should acquaint themselves with this clinical scenario because such patients present first to them and not to hematologists.Pleural effusion has been reported in association with solid tumors and sometimes as their presenting sign. A multivariate analysis by Faiz et al., Leukemia Lymphoma (2012) between 1997 and 2007 showed that 111 patients with acute leukemias had pleural effusions and the median overall survival in these patients was shorter than those with absence of pleural effusions. Other studies revealed rare occurrences of pleural effusion in AML patients.

The authors need to highlight salient features of previously reported patients and compare them with their patient hoping to find predictive factors for development of such rare clinical scenarios and define better treatments.

I have read this submission. I believe that I have an appropriate level of expertise to confirm that it is of an acceptable scientific standard.

# Peer review: Referee response for version 1

**Published:** 2012-11-22

**Authors:** Stephen Nimer

**Affiliations:** 1 Memorial Sloan-Kettering Cancer Center, New York, NY, USA

This article points out an unusual clinical presentation of acute myeloid leukemia (AML) that illustrates the heterogeneity of presentations of extramedullary disease.

While the diagnosis was clear at the onset, the patient had 64% circulating blasts and the pleural effusion was unusual and required evaluation. As monocyte subtypes of AML have a propensity for tissue invasion and the core binding factor (CBF) leukemia can be associated with granule cystic sarcomas, a bit more information on the immunophenotype of the AML would have been helpful in assessing this case. Also, given the many molecular markers that define the prognosis of AML patients, more details on any molecular studies that were done, or could have been done, would be helpful.

I have read this submission. I believe that I have an appropriate level of expertise to confirm that it is of an acceptable scientific standard.

# Peer review: Referee response for version 1

**Published:** 2012-11-21

**Authors:** Sharon Savage

**Affiliations:** 1 Division of Cancer Epidemiology & Genetics, National Cancer Institute, National Institutes of Health (NIH), Bethesda, MD, USA

This case report describes a case of acute myeloid leukemia (AML) that presented with a large pleural effusion. This unusual presentation is important to recognize when evaluating patients with pleural effusions. This particular case report illustrates the importance of a cytopathology evaluation.

The report itself would benefit from an updated reference on chromosomal abnormalities in AML, and it would also be helpful to know the doses of the chemotherapeutic agents and if the patient was treated on a clinical trial for newly diagnosed AML.

I have read this submission. I believe that I have an appropriate level of expertise to confirm that it is of an acceptable scientific standard.
